# Standard-of-care ciltacabtagene autoleucel in earlier versus later lines of therapy for relapsed or refractory multiple myeloma: a nationwide registry analysis

**DOI:** 10.1186/s13045-026-01806-6

**Published:** 2026-05-20

**Authors:** Nico Gagelmann, Hermann Einsele, Sarah Flossdorf, Samih Smaili, Klaus Metzeler, Christoph Scheid, Lars Bullinger, Sandra Sauer, Marc Raab, Justus Duyster, Hans Christian Reinhardt, Raphael Teipel, Winfried Alsdorf, Claudia Lengerke, Wolfgang Herr, Natalie Schub, Francis Ayuketang Ayuk, Florian Bassermann, Christian Schultze-Florey, Roland Schroers, Mathias Hänel, Nicola Giesen, Ahmet Elmaagacli, Andreas Burchert, Thomas Weber, Dimitrios Mougiakakos, Stefan Knop, Fabian Müller, Nicolaus Kröger, Maximilian Merz

**Affiliations:** 1 University Medical Center Hamburg-Eppendorf, Hamburg, Germany; 2 Universitätsklinikum Würzburg, Würzburg, Germany; 3 Universitätsklinikum Essen, Essen, Germany; 4 German Registry for Hematopoietic Stem Cell Transplantation and Cell Therapy, Ulm, Germany; 5 Universitätsklinikum Leipzig, Leipzig, Germany; 6 Universitätsklinikum Köln, Köln, Germany; 7 Charité - Universitätsmedizin Berlin, Berlin, Germany; 8 Universitätsklinikum Heidelberg, Heidelberg, Germany; 9 Universitätsklinikum Freiburg, Freiburg, Germany; 10 Universitätsklinikum Carl Gustav Carus a. d. TU Dresden, Dresden, Germany; 11 Universitätsklinikum Tübingen, Tübingen, Germany; 12 Universitätsklinikum Regensburg, Regensburg, Germany; 13 Universitätsklinikum Schleswig-Holstein / Campus Kiel, Kiel, Germany; 14 TUM Universitätsklinikum, Klinikum rechts der Isar, Munich, Germany; 15 Hämatologie, Hämostaseologie, Onkologie und Stammzelltransplantation, Medizinische Hochschule Hannover (MHH), Hannover, Germany; 16 Knappschaft Kliniken Universitätsklinikum Bochum, Bochum, Germany; 17 Klinikum Chemnitz gGmbH, Chemnitz, Germany; 18 Robert-Bosch-Krankenhaus, Stuttgart, Germany; 19 Asklepios Klinik St. Georg, Hamburg, Germany; 20 Univ.-Klinikum Gießen und Marburg GmbH, Marburg, Germany; 21 Universitätsklinikum Halle (Saale), Halle, Germany; 22 Universitätsklinikum Magdeburg, Magdeburg, Germany; 23 Klinikum Nürnberg Nord, Nürnberg, Germany; 24 Universitätsklinikum Erlangen, Erlangen, Germany; 25 Fraunhofer Institute for Immunology and Cell Therapy, Leipzig, Germany; 26 Myeloma and Cellular Therapy Services, Memorial Sloan Kettering Cancer Center, 1275 York Ave, New York, NY 10065 USA

**Keywords:** Multiple myeloma, Chimeric antigen receptor T cells, Ciltacabtagene autoleucel

## Abstract

**Background:**

Ciltacabtagene autoleucel (cilta-cel) is a BCMA-directed chimeric antigen receptor (CAR) T-cell therapy approved for relapsed or refractory multiple myeloma (RRMM). Following the CARTITUDE-1 results in heavily pretreated patients, the randomized phase 3 CARTITUDE-4 trial demonstrated superior progression-free survival (PFS) and overall survival for cilta-cel compared with standard of care in lenalidomide-refractory patients after one to three prior lines, leading to label expansion in 2024. However, real-world data characterizing outcomes in this earlier-line indication are lacking.

**Methods:**

We analyzed all patients with RRMM receiving standard-of-care cilta-cel between 2022 and 2025 from the German Registry for Stem Cell Transplantation and Cellular Therapy. Patients were stratified by prior lines of therapy into an Early group (1–3 prior lines) and a Late group (> 3 prior lines). The primary endpoint was PFS. Secondary endpoints included overall response rate, response conversion, and safety outcomes including cytokine release syndrome, immune effector cell-associated neurotoxicity syndrome (ICANS), non-ICANS neurotoxicity, and non-relapse mortality. Prognostic associations were assessed using restricted cubic spline Cox regression and univariable Cox models.

**Results:**

Of 606 patients, 177 (30%) were treated in the Early and 429 (70%) in the Late setting. The overall response rate was 91% and 88%, with complete response in 63% and 54%, respectively. The 12-month PFS was 79% for Early and 70% for Late cilta-cel. Depth of response was the strongest predictor of PFS in both cohorts, with patients maintaining complete response showing 100% PFS at 12 months irrespective of treatment line. Extramedullary disease was adversely prognostic in both groups, whereas high-risk cytogenetics were not associated with inferior PFS in the Early group. Non-ICANS neurotoxicity occurred less frequently in the Early group (3% versus 8%), while non-relapse mortality was comparable (6% versus 7%).

**Conclusions:**

This analysis demonstrates that cilta-cel in earlier lines of therapy achieves deep responses and high PFS consistent with the CARTITUDE-4 trial. These results provide real-world evidence for the deployment of cilta-cel as early as first relapse and may be a benchmark outside prospective trials.

**Supplementary Information:**

The online version contains supplementary material available at 10.1186/s13045-026-01806-6.

## Introduction

Ciltacabtagene autoleucel (cilta-cel) is a B-cell maturation antigen (BCMA)-directed chimeric antigen receptor (CAR) T-cell therapy that has transformed the treatment of relapsed or refractory multiple myeloma (RRMM). Based on results from the single-arm, phase 1b/2 CARTITUDE-1 study, which demonstrated deep and durable responses in heavily pretreated patients,^1–3^ cilta-cel received regulatory approval in 2022 by both the United States Food and Drug Administration and the European Medicines Agency for patients who had received three or more prior lines of therapy including a proteasome inhibitor, an immunomodulatory drug, and an anti-CD38 antibody.

Subsequently, the randomized phase 3 CARTITUDE-4 trial demonstrated superior progression-free survival and, at longer follow-up, an overall survival benefit for cilta-cel compared with standard-of-care regimens in lenalidomide-refractory patients after one to three prior lines of therapy [1, 2]. CARTITUDE-4 also suggested a favorable safety profile in earlier lines, potentially attributable to more effective bridging therapy and lower disease burden at the time of infusion [3]. Based on these data, both the FDA and EMA expanded the indication for cilta-cel in 2024 to include patients after at least one prior line of therapy who are refractory to lenalidomide.

Real-world analyses have confirmed trial outcomes and demonstrated superior results with cilta-cel compared with idecabtagene vicleucel (ide-cel) in the heavily pretreated CARTITUDE-1 indication [4–6] However, no real-world data are currently available for patients treated in the earlier-line CARTITUDE-4 indication. We therefore leveraged a national real-world registry to describe efficacy and safety of cilta-cel in early- and later-line indication and to identify outcome patterns particularly in early-line cilta-cel.

## Methods

### Study design

This is a real-world analysis of the German Registry for Stem Cell Transplantation and Cellular Therapy. The study included all documented adult patients (≥ 18 years) with RRMM who received cilta-cel as a commercially available product between 2022 and 2025. Patients were stratified into two groups based on the number of prior lines of therapy received before cilta-cel infusion: an Early line group (1–3 prior lines) and a Late line group (> 3 prior lines). This categorization was chosen to reflect the evolving therapeutic landscape, where cilta-cel is increasingly used in earlier treatment lines following regulatory approval in the 1–3 prior lines setting (CARTITUDE-4), in addition to its established indication in heavily pretreated patients (> 3 prior lines, CARTITUDE-1). The study was approved by the DRST board and conducted in accordance with the Declaration of Helsinki.

### Endpoints and definitions

The primary endpoint was progression-free survival (PFS), defined as the time from cilta-cel infusion to disease progression or death from any cause, whichever occurred first. Patients without an event were censored at the date of last follow-up. Secondary endpoints included overall response rate (ORR), response conversion (defined as evolution in disease status from before to after infusion), and safety outcomes including cytokine release syndrome (CRS), immune effector cell-associated neurotoxicity syndrome (ICANS),^10^ non-ICANS neurotoxicity (NINT), infectionsn and non-relapse mortality. Disease status before infusion and best response were classified as complete response (CR), very good partial response (VGPR), partial response (PR), or less than partial response (< PR) according to International Myeloma Working Group (IMWG) criteria [7]. CRS and ICANS were graded per American Society for Transplantation and Cellular Therapy consensus criteria [8]. High-risk cytogenetics was defined as the presence of at least one of the following: t(4;14), del(17p), gain(1q), or t(14;16).

### Statistical analysis

Baseline characteristics were summarized for continuous variables using the Mann-Whitney U test, and categorical variables were compared using the Fisher’s exact test. Progression-free survival was estimated using the Kaplan-Meier method with 95% confidence intervals (CI). Landmark PFS rates were reported at 6 and 12 months.

The association between the number of prior therapy lines (as a continuous variable) and PFS was modeled within each group using a Cox proportional hazards regression in which the predictor was entered as a restricted cubic spline with four knots at the 5th, 35th, 65th, and 95th percentiles, allowing the hazard of progression or death to vary smoothly and non-linearly across the observed range of the predictor. The median value of the predictor within each group was used as the reference (hazard ratio = 1 by construction at that point), and the curves in Fig. 3A therefore represent the relative risk of progression or death at any given number of prior lines (left panel) or any given diagnosis-to-infusion interval (right panel) compared with a patient at the median value of that predictor in the same group. An analogous model was fitted for the diagnosis-to-infusion interval. Hazard ratios and 95% confidence intervals were derived from the fitted spline at a grid of predictor values and are displayed on a logarithmic axis.

Univariable Cox regression analyses were performed within each group (Early and Late) to assess the prognostic impact of baseline covariates on PFS. The following variables were evaluated based on clinical relevance and prior evidence: age at infusion (per year), diagnosis-to-infusion interval (per month), sex (female vs. male), extramedullary disease (EMD; present vs. absent), ECOG performance status (1 vs. 0, > 1 vs. 0), International Staging System (ISS; stage II vs. I, stage III vs. I), high-risk cytogenetics (present vs. absent), individual cytogenetic abnormalities (t(4;14), del(17p), gain(1q), t(14;16); each present vs. absent), prior autologous hematopoietic cell transplantation (yes vs. no), and turnaround time from apheresis collection to infusion (per day).

Given the non-randomized registry design with clinically determined treatment allocation, the hypothesis-generating nature of this analysis, and the expected modest sample size of the Early group, formal statistical comparisons for outcomes between groups were intentionally omitted to avoid the risk of spurious inference from multiple testing. All analyses were done using R statistical software version 4.3.3.

## Results

**Fig. 1:**
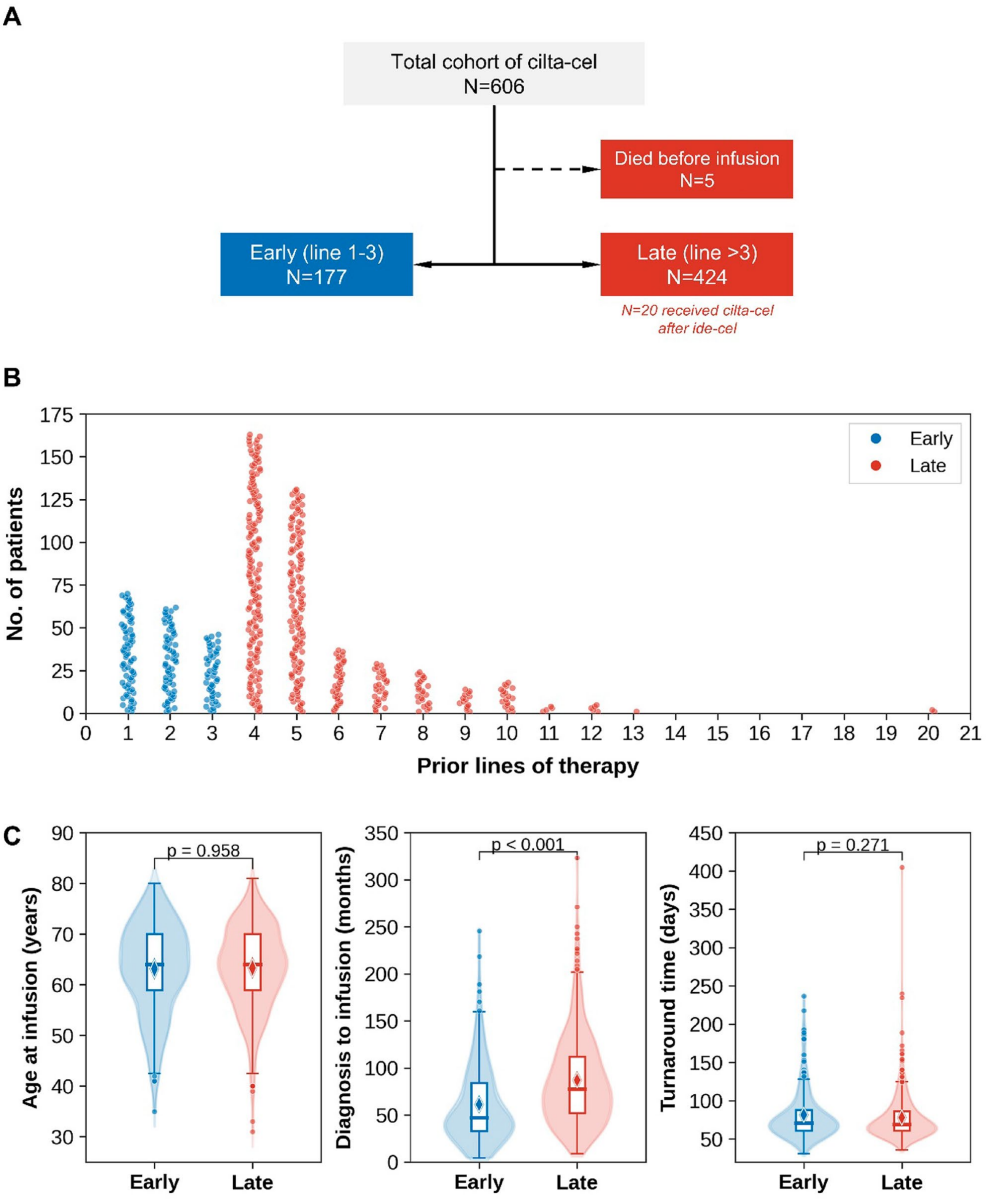
Patient disposition and baseline characteristics. (**A**) Flow diagram of the total cilta-cel cohort (N = 606). Patients were stratified into the Early group (1–3 prior lines of therapy; n = 177) and the Late group (> 3 prior lines; n = 429). Twenty patients had received prior idecabtagene vicleucel (ide-cel), and 5 patients died before infusion in the Late group, leaving 424 infused patients. (**B**) Distribution of prior lines of therapy in the Early (blue) and Late (red) groups. Each dot represents an individual patient. (**C**) Comparing of Early and Late groups for age at infusion (left; p = 0.958), time from diagnosis to infusion (center; p < 0.001), and turnaround time from leukapheresis to infusion (right; p = 0.271). Box plots display median (horizontal line), interquartile range (box), and range (whiskers). Individual data points are overlaid. P values were calculated using the Wilcoxon rank-sum test

Of the total cohort of 606 patients, 177 patients (30%) had received 1–3 prior lines of therapy (Early group, median of 2 prior lines), and 429 patients (70%) had received > 3 prior lines (Late group, median of 5 lines; Fig. 1A-B). Within the Late group, 5 patients died between apheresis and infusion. The median time from diagnosis to cilta-cel infusion was shorter in the Early group, while the turnaround time from apheresis to cilta-cel infusion was similar between groups (median 71 days in the Early versus 69 days in the Late group; *P* = 0.27; Fig. 1C).

The median age was 64 years in both groups (Fig. 1C), male sex was similarly distributed, and most had ECOG performance status of 0 or 1 (Table 1). Extramedullary disease (EMD) and ISS stage III was more common in the Late group (28% and 46% versus 20% and 40%), while by contrast high-risk cytogenetic abnormalities were observed in 47% of the Early and 37% of the Late group.

**Table 1:** Patient characteristics

Characteristic	Early cilta-cel (*n* = 177)	Late cilta-cel (*n* = 429)
**Sex**,** no. (%)**		
Male	103 (58)	247 (58)
Female	75 (42)	182 (42)
**ECOG**,** no. (%)**		
0	70 (40)	173 (43)
1	89 (51)	190 (47)
2	10 (6)	34 (8)
3	6 (3)	9 (2)
Unknown	2	23
**ISS**,** no. (%)**		
I	50 (31)	85 (23)
II	46 (29)	119 (32)
III	63 (40)	172 (46)
Unknown	18	53
**Extramedullary disease**,** no. (%)**		
No	96 (80)	206 (73)
Yes	24 (20)	78 (27)
Unknown	57	145
**High-risk cytogenetics**,** no. (%)**		
No	83 (53)	206 (63)
Yes	73 (47)	121 (37)
Unknown	21	102
**Prior cellular therapy**,** no. (%)**		
Autologous transplantation	126 (71)	295 (69)
Ide-cel	0	20 (5)

ISS, International Staging System; no, number

### Efficacy

**Fig. 2:**
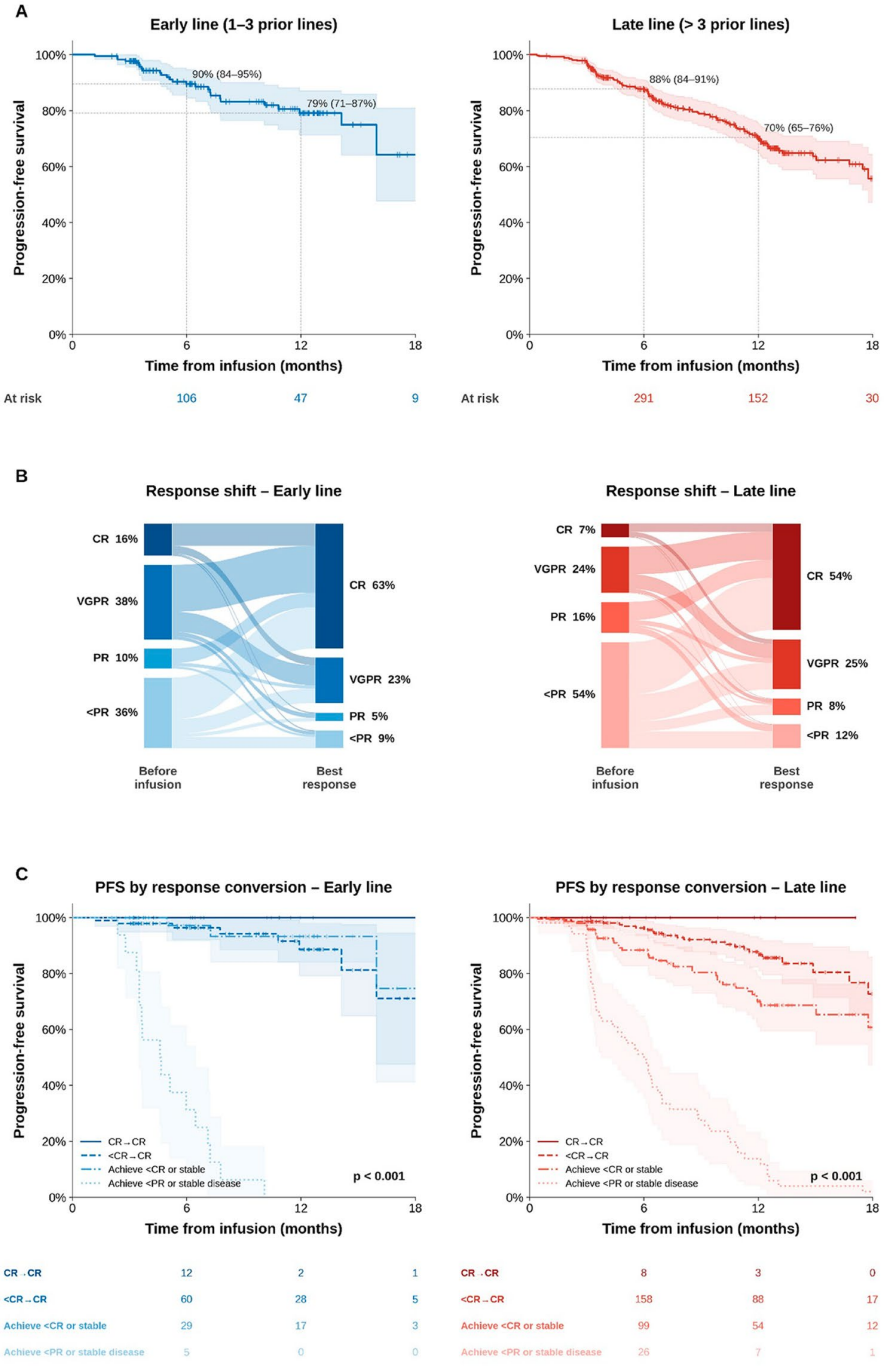
Efficacy outcomes by treatment line. (**A**) Kaplan-Meier estimates of progression-free survival (PFS) for the Early group (1–3 prior lines; left) and the Late group (> 3 prior lines; right). The 6-month and 12-month PFS rates with 95% confidence intervals are annotated. Shaded areas represent 95% confidence intervals. (**B**) Alluvial plots depicting the depth of response before cilta-cel infusion (left bar) and best response after infusion (right bar) for the Early group (left panel) and the Late group (right panel). Response categories include complete response (CR), very good partial response (VGPR), partial response (PR), and less than partial response (< PR). (**C**) PFS stratified by response conversion category in the Early group (left) and the Late group (right). Patients were categorized as: CR→CR (maintained CR), < CR→CR (deepened to CR), achieved < CR or stable response, and achieved < PR or stable disease. Numbers at risk are shown below each plot. P values were calculated using the log-rank test

The median follow-up was 7.2 months (95% CI, 6.4–10.1 months) for the Early and 11.0 months (95% CI, 8.8–11.9 months) for the Late group. In the Early group, the 6-month PFS rate was 90% (95% CI, 84–95%) and the 12-month PFS rate was 79% (95% CI, 71–87%; Fig. 2A). Among Late patients, the 6-month PFS rate was 88% (95% CI, 84–91%) and the 12-month PFS rate was 70% (95% CI, 65–76%), with a median PFS of 21.2 months.

Prior to infusion, more Late patients presented with advanced disease states (Fig. 2B): 54% had a disease status of less than PR, compared with 36% in the Early group. Following cilta-cel infusion, the ORR was 91% in the Early and 88% in the Late group, with a CR as best response in 63% and 54%, respectively. In both cohorts, a substantial proportion of patients with pre-existing VGPR or PR converted to CR, underscoring the ability of cilta-cel to deepen responses across a wide spectrum of baseline disease states.

The prognostic impact of response depth on PFS was pronounced in both groups (log-rank *P*<0.001 for both), with similar effect gradients (Fig. 2C). Patients who maintained CR throughout cilta-cel therapy, the 12-month PFS was 100% in both Early and Late groups. In contrast, patients who went into cilta-cel therapy with less than PR and never achieved a response showed a 12-month PFS of 0% in the Early group and 14% (95% CI, 4–23%) in the Late group, with a median PFS of only 4.6 and 6 months, respectively. Patients with disease in response before cilta-cel infusion and who maintained or improved their response status showed an intermediate PFS profile, with 6-month PFS of 96% for those improving their response status to CR and 97% for those who improved their response status and maintained response (less than CR) in the Early group. Outcomes for those patients appeared more gradual in the Late group, showing 6-month PFS of 96% and 88%, respectively.

### Correlates with progression-free survival

**Fig. 3:**
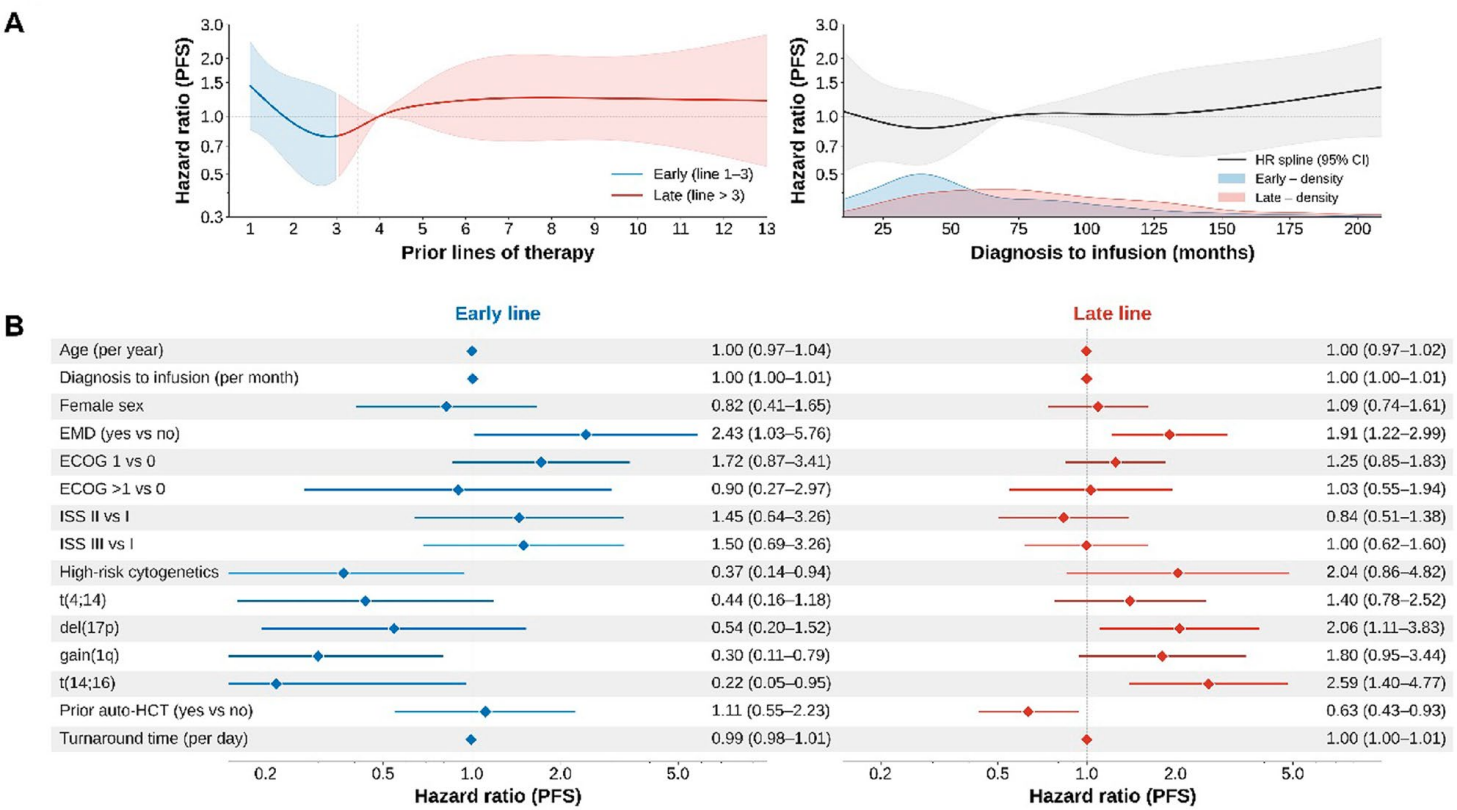
Subgroup analysis of progression-free survival. (**A**) Left panel: restricted cubic spline analysis of the risk of progression or death (hazard ratio, HR) for PFS as a continuous, potentially non-linear function of the number of prior lines of therapy. At each value of the predictor, the curve represents the relative risk of progression or death compared with a patient at the median number of prior lines in the same group (reference, HR = 1, marked by the horizontal dashed line); the shaded band is the 95% confidence interval. The HR is plotted on a logarithmic scale. Right panel: analogous spline for the interval from diagnosis to infusion (months), with density distributions overlaid for the Early (blue) and Late (red) groups. (**B**) Forest plots of Cox proportional hazards regression for subgroups in the Early group (left) and the Late group (right). Point estimates are represented by diamonds, and horizontal lines indicate 95% confidence intervals. The dashed vertical line at HR = 1.0 represents no effect

We then aimed to describe potential correlates with outcome in patients receiving standard-of-care cilta-cel. Figure 3A displays the restricted cubic spline models, in which the curve represents the estimated risk of progression or death at any given value of the predictor relative to a reference patient at the median value in the same group; the two groups showed distinct patterns. In the Early setting, patients who had received a higher number of prior lines within the 1-3-line range had a progressively lower risk of progression or death than patients at the median number of prior lines, a pattern most plausibly reflecting selection of patients with primary-refractory disease and early relapse into the Early-line cohort rather than a genuine protective effect of additional pretreatment. In the Late group, the risk of progression or death remained approximately flat across the observed range of prior lines, with confidence bands overlapping unity throughout. The diagnosis-to-infusion interval showed no clinically meaningful association with PFS in either group: the estimated risk remained close to that of the reference patient across the full range of observation, with confidence bands spanning unity (Fig. 3A, right panel).

We next used univariable Cox regression within each group to describe the prognostic landscape of baseline covariates (Fig. 3B); hazard ratios (HR) are reported in the forest plot and in the text below. Extramedullary disease was associated with a higher risk of progression or death in both groups (Early: HR 2.43; Late: HR 1.91). The cytogenetic pattern differed between the groups. In the Early-line cohort, individual high-risk cytogenetic abnormalities were not associated with an increased risk of progression or death, and both gain(1q) (HR 0.30) and t(14;16) (HR 0.22) were associated with a lower risk relative to their absence; the composite high-risk cytogenetic variable likewise showed a lower hazard in Early-line patients (HR 0.37), a finding that may reflect the biological context of earlier treatment and is consistent with observations from CARTITUDE-4. In the Late-line cohort, del(17p) (HR 2.06) and t(14;16) (HR 2.59) were associated with a higher risk of progression or death, consistent with their established role as high-risk features in RRMM. Prior autologous stem-cell transplantation was associated with a lower risk of progression or death in the Late group (HR 0.63) but not in the Early group, potentially reflecting a more favourable disease biology or a more intensively pretreated but still transplant-eligible subset. Age, sex, ECOG performance status, ISS stage, and apheresis-to-infusion turnaround time were not associated with PFS in either group.

In a sensitivity analysis stratified by cumulative number of high-risk cytogenetic abnormalities (HRCA: 0 vs. 1 vs. ≥ 2), each additional HRCA was associated with increased PFS hazard in the Late group (HR 1.23 [95% CI, 1.02–1.48]) but with reduced hazard in the Early group (HR 0.59 [95% CI, 0.36–0.95]), where patients with ≥ 2 HRCAs achieved a 12-month PFS of 90% (95% CI, 66–98%).

Furthermore, to identify functional high-risk (FHR) patients across both cohorts, we fitted group-specific probabilistic models using baseline clinical and biological features, calibrated to published benchmarks (Supplementary Methods). Under this approach, 59 of 177 Early-line patients (33%) and 64 of 424 Late-line patients (15%) were classified as FHR, both subgroups showing the expected enrichment for adverse features including high-risk cytogenetics, ISS III, extramedullary disease, and refractory disease status at infusion. In the Early group, the 6-month PFS was 86% in FHR versus 91% in non-FHR patients, and the 12-month PFS was 74% versus 82% (Supplementary Fig. S1), respectively (HR 1.35 [95% CI, 0.63–2.90]). In the Late group, FHR patients showed a significant residual prognostic penalty, with 6-month PFS of 81% versus 89% and 12-month PFS of 63% versus 71% in non-FHR patients (HR 1.69 [95% CI, 1.06–2.70]; Supplementary Fig. S1).

We next evaluated impact of bridging, and of the 159 patients (26%) with reliably documented regimen-level bridging therapy, proteasome-inhibitor-based triplets (most commonly carfilzomib-containing) were the predominant backbone in both cohorts (49%), with anti-CD38 antibody-containing regimens (35%), IMiD-containing regimens (31%), and elotuzumab-containing regimens (15%) also commonly used. Talquetamab-containing bridging was administered in 35 patients (6% of the overall cohort), only in the Late-line setting (Supplementary Table S1). PFS did not differ meaningfully between bridged patients and the overall cohort in either group (6-month PFS 87% in both the Early and Late bridging subgroups; 12-month PFS 79% and 70%, respectively), and outcomes in the talquetamab-bridging subgroup (6-month PFS 82%, 12-month PFS 69%) were comparable to those of the overall Late-line bridging cohort (Supplementary Fig. S2).

### Safety

**Fig. 4:**
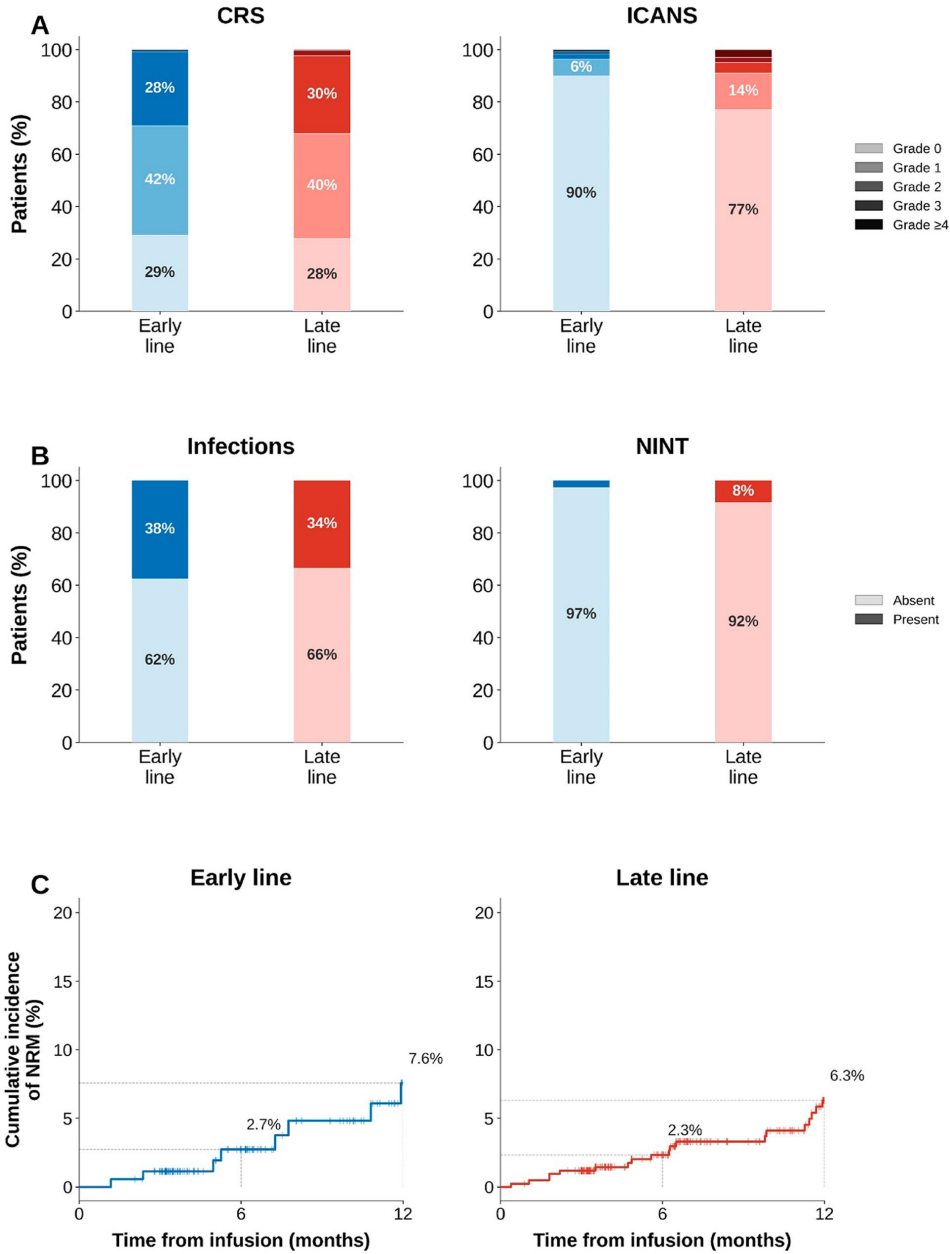
Safety profile by treatment line. (**A**) Grade distribution of cytokine release syndrome (CRS; left) and immune effector cell-associated neurotoxicity syndrome (ICANS; right) in the Early and Late groups. (**B**) Incidence of infections (left) and non-ICANS neurotoxicity (NINT; right) in the Early and Late groups. Stacked bars represent the proportion of patients with (present) or without (absent) the respective event. (**C**) Cumulative incidence of non-relapse mortality (NRM) by line of therapy

The safety profile of cilta-cel was broadly comparable between the two groups. In terms of CAR-T-specific adverse events, grade 1 CRS was the most frequently observed severity, occurring in 42% and 40% of evaluable patients in the Early and Late groups, respectively. Grade 2 CRS occurred in 28% versus 30%, and grade ≥ 3 events were uncommon (Fig. 4A). For neurotoxicity, grade ≥ 2 ICANS was rare, occurring in 4% of early-line and 2% of late-line patients. One fatal grade 5 ICANS event was recorded in the Late cohort.

Non-ICANS neurotoxicity was reported in a total of 22 cases, accounting for 3% of Early and 8% of Late patients (Fig. 4B). The NINT cases presented with a broad phenotypic spectrum, though facial nerve involvement emerged as the most frequent manifestation, with cranial neuropathy (predominantly peripheral facial palsy) documented in approximately one third of cases (Table 2). A distinct subgroup presented with peripheral nervous system involvement, including polyneuropathy, Guillain-Barré syndrome (*n* = 2), and sensorimotor disturbances of the extremities. Central nervous system events, while less common, were the most severe: these included one case each of autoimmune cerebellitis/brainstem encephalitis, progressive multifocal leukoencephalopathy, CAR-T-cell-mediated encephalopathy with concurrent cerebellar stroke, and one case of dissociative amnesia with vertigo. The remainder presented with less localizing manifestations (fatigue, gait disturbance, confusion, and mood or sleep disturbance) that nonetheless required clinical attention. Taken together, the NINT spectrum observed were consistent with an immune-mediated, predominantly post-acute process with cranial nerve predilection.

**Table 2:** Non-ICANS neurotoxicity cases

#	Group	Manifestation	Category	Max grade	Timing
1	Early	Guillain-Barré syndrome	PNS	4	Day 100
2	Early	Autoimmune cerebellitis / brainstem encephalitis	CNS	3	Day 100
3	Early	Progressive multifocal leukoencephalopathy	CNS	NR	Day 100
4	Late	Parkinson syndrome	CNS	3	Day 100
5	Late	Facial nerve palsy	Cranial	3	Day 100
6	Late	Facial nerve palsy	Cranial	Unknown	Day 100
7	Late	Facial nerve palsy	Cranial	Unknown	Day 100
8	Late	Facial paresis	Cranial	2	Day 100
9	Late	Facial paralysis	Cranial	Unknown	Day 100
10	Late	Facial paralysis; gait disturbance with severe polyneuropathy	Cranial/PNS	Unknown	Day 100
11	Late	Idiopathic peripheral facial paresis	Cranial	2	Day 100
12	Late	Facial paresis	Cranial	2	Day 100
13	Late	Recurrent dizziness; intermittent hypaesthesias; known PNP; dysgeusia; dysphagia; depressive mood; insomnia	PNS/Other	NR	Day 100
14	Late	Polyneuropathy left foot	PNS	NR	Day 100
15	Late	Guillain-Barré syndrome	PNS	5 (fatal)	Month 6
16	Late	CAR-T-mediated encephalopathy; right cerebellar stroke	CNS	3	Day 100
17	Late	Dissociative amnesia; vertigo	CNS	3	Day 100
18	Late	Gait disorder; confusion	CNS	Unknown	Day 100
19	Late	Persistent pronounced fatigue	Other	Unknown	Day 100
20	Late	Fatigue	Other	Unknown	Day 100
21	Late	Hypertensive crisis	Other	3	Day 100
22	Late	Late-onset confusion	Other	2	Day 100

PNS, peripheral nervous system; CNS, central nervous system; NR, not reported; PNP, peripheral neuropathy

In terms of NRM, a total of 31 events were observed across the entire cohort. The cumulative incidence rates of 2.7% at 6 months and 7.6% at 12 months in the Early group and 2.3% at 6 months and 6.3% at 12 months in the Late group (Fig. 4C). The comparable NRM across treatment lines, which are in line with CARTITUDE trials, supports the safety and feasibility of commercial cilta-cel administration in the real-world setting across both early and late lines of therapy.

Immune effector cell-associated hemophagocytic lymphohistiocytosis-like syndrome (IEC-HS) was documented in 3 of 357 evaluable patients (0.8%; 1 Early, 2 Late), all with preceding grade 2 CRS and with onset spanning day-100 to month-12; acknowledging that coding might have predated the ASTCT consensus criteria and was performed locally without central re-adjudication and should therefore be interpreted with caution. Two patients were alive at last follow-up and one fatal case in the Late-line cohort occurred.

### Impact of prior CAR-T cell therapy

**Fig. 5:**
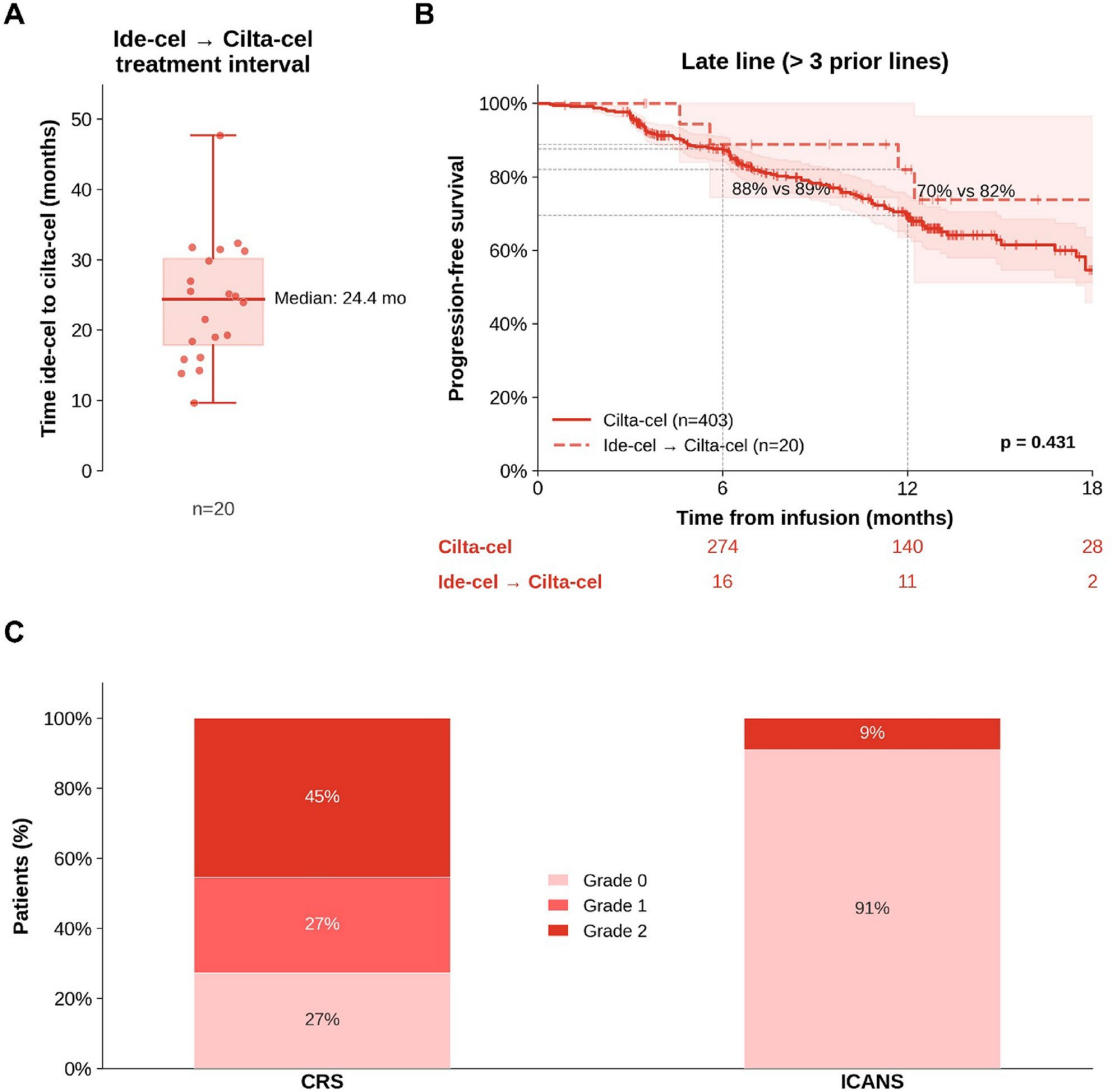
Outcomes in patients of the Late group with prior ide-cel exposure. (**A**) Treatment interval from ide-cel to cilta-cel infusion (months) in the 20 patients who received sequential CAR-T therapy. Each dot represents an individual patient; the median interval is annotated. (**B**) Kaplan-Meier estimates of PFS in the Late group comparing patients who received cilta-cel without prior ide-cel (solid line) and patients who received ide-cel followed by cilta-cel (dashed line; n = 20). The 6-month and 12-month PFS rates are annotated for both subgroups. The p value was calculated using the log-rank test. (**C**) Left: Grade distribution of CRS in the ide-cel→cilta-cel subgroup, displayed as a stacked bar chart with grades 0, 1, and 2. Right: grade distribution of ICANS

Within the Late group, 20 patients (5%) had received a prior anti-BCMA CAR-T cell infusion with ide-cel before cilta-cel, with a median interval from ide-cel infusion to cilta-cel infusion of 24.4 months, with individual intervals ranging from approximately 10 to 48 months (Fig. 5A).

Despite prior CAR-T exposure targeting the same antigen, cilta-cel appeared to demonstrate efficacy in this subgroup. The 6-month PFS rate was 89% (95% CI, 74–100%) and the 12-month PFS rate was 82% (95% CI, 63–100%), which appeared similar compared with CAR-T-naive patients (Fig. 5B**)**.

Regarding toxicity in the ide-cel-exposed subgroup, grade 1 CRS occurred in 27%, grade 2 in 45%, and no patient experienced grade ≥ 3 CRS (Fig. 5C). 91% experienced no ICANS and one patient (9%) developed grade 2 ICANS. These findings suggest no increased CAR-T-specific toxicity profile for cilta-cel after ide-cel.

## Discussion

This first nationwide analysis of standard-of-care cilta-cel demonstrates robust efficacy across both earlier-line and later-line settings. The Early group achieved a 12-month PFS rate of 79%, while the Late group reached 70% with a median PFS of 21.2 months - findings that align closely with the respective pivotal trials: CARTITUDE-4 reported a 12-month PFS rate of 76% in patients with one to three prior lines, and long-term follow-up of CARTITUDE-1 demonstrated a median PFS of 34.9 months in heavily pretreated patients [1, 2, 9–11]. Although these two real-world populations differ fundamentally in disease biology, treatment history, and selection mechanisms - precluding direct comparison between them - these results confirm that the efficacy of cilta-cel translates reliably from controlled trial settings into routine clinical practice.

The distinction between both cohorts is underscored by analysis of prior therapy lines and the interval from diagnosis to CAR T-cell infusion. Within the Early group, we showed that increasing lines of prior therapy were paradoxically associated with a lower hazard of progression, most likely reflecting patient selection, indicating that patients referred for cilta-cel after only one line and with a short duration from primary diagnosis to CAR T-cell infusion represent a functionally high-risk population with early relapse after frontline therapy [12]. In the current era of highly effective quadruplet induction regimens, early lenalidomide-refractory relapse identifies a population with intrinsically aggressive disease biology. Meanwhile, the diagnosis-to-infusion interval showed no significant association with PFS especially in the Late group, suggesting that this variable, which inherently introduces immortal time bias favoring patients with prolonged treatment courses, is not a reliable prognostic factor in this context.

Several biological mechanisms may support the favorable outcomes observed with earlier-line cilta-cel. Correlative analyses from the CARTITUDE-1 and CARTITUDE-4 trials have demonstrated that patients with fewer prior lines of therapy retain higher baseline levels of CD4-positive naïve T cells, which are significantly associated with prolonged PFS. Furthermore, tumor microenvironment profiling from CARTITUDE-4 showed that patients with one or two prior lines exhibited a more immunocompetent bone marrow milieu, characterized by elevated costimulatory molecules, enhanced antigen-presenting capacity, and greater T-cell receptor activation at baseline [13]. These findings suggest that earlier application of cilta-cel may capitalize on preserved immune fitness, enabling more robust CAR T-cell expansion and sustained antitumor immunity.

Beyond immune fitness, the availability of more effective bridging therapy options in earlier lines likely contributes to both efficacy and safety outcomes [14]. In our study, a higher proportion of patients in the Early group achieved at least a partial response at the time of infusion compared to the Late group, reflecting the diminishing effectiveness of available bridging regimens with increasing lines of prior therapy. In addition, response conversion from pre-infusion disease status to CR was a dominant driver of PFS across the entire cohort, consistent with a recent analysis demonstrating that remission conversion is the principal determinant of CAR T-cell therapy outcomes in RRMM [6]. Data from CARTITUDE-4 have corroborated this principle: a post-hoc analysis showed that deeper bridging responses correlated with improved survival and safety, and notably, none of the patients who achieved at least a partial response to bridging developed immune effector cell-associated parkinsonism [3]. The clinical implications are clear: optimizing disease control before CAR T-cell infusion through effective bridging is not merely supportive but therapeutic.

The safety data from our analysis align with these observations. Non-ICANS neurotoxicities were observed in 3% of Early and 8% of Late patients, a pattern that mirrors the reduction in parkinsonism from 6% in CARTITUDE-1 to less than 1% in CARTITUDE-4. In both the trial and our real-world setting, this finding is plausibly linked to lower tumor burden at infusion and reduced inflammatory drive [15], as high tumor burden, high-grade CRS, and excessive CAR T-cell expansion have been identified as associated factors for movement and neurocognitive treatment-emergent adverse events [16–20]. The phenotypic spectrum of NiNTs in our cohort, predominantly cranial neuropathies and peripheral nervous system involvement, was consistent with an immune-mediated, post-acute process and aligns with the known toxicity profile of cilta-cel [21].

Among prognostic factors, EMD was consistently associated with inferior PFS in both the Early and Late groups, underscoring that this feature identifies a biologically distinct and treatment-resistant subset regardless of treatment line [22–24]. This finding has immediate clinical relevance, as it highlights the need for novel strategies in patients with extramedullary involvement, including dual-targeting approaches [25, 26] incorporating GPRC5D [27]- or FcRH5-directed [28] agents either as bridging [29, 30], consolidation, or maintenance therapy after cilta-cel. By contrast, the cytogenetic risk profile showed distinct patterns across the two groups. In the Early group, high-risk cytogenetics as a composite variable, and gain(1q) individually, were not associated with adverse outcomes and in fact appeared to be associated with a lower hazard, a finding that aligns with the subgroup analyses from CARTITUDE-4. This observation may reflect that the accumulation of 1q21 gain or even amplification during the natural history of RRMM is associated with increasingly proliferative disease biology [31] and that earlier intervention with cilta-cel may precede the clonal evolution that renders this abnormality prognostically unfavorable. In the Late group, del(17p) and t(14;16) retained their established adverse prognostic significance.

In an exploratory model-based analysis addressing FHR disease, predicted FHR patients in the Late-line cohort showed a numerically inferior 12-month PFS compared with non-FHR patients (63% vs. 71%), whereas for the Early group PFS was 74% vs. 82%. These observations are hypothesis-generating and should be interpreted in light of the model-derived nature of the FHR classification. Taken together with the post-hoc subgroup findings from CARTITUDE-1 and the real-world observations,^12^ they are consistent with – but do not establish – a biological model in which earlier cilta-cel deployment intersects with functionally aggressive disease before the proliferative, multi-refractory phenotype has fully evolved.

An emerging clinical question is whether patients who relapse after an initial CAR T-cell therapy can benefit from a second BCMA-directed product. Within the Late group, 20 patients had received prior ide-cel before cilta-cel, and this subgroup demonstrated encouraging outcomes and no evidence of increased CAR T-specific toxicity. Notably, no cases suggestive of biallelic BCMA loss or primary refractoriness were documented. These data support the concept that sequential BCMA-directed CAR T-cell therapy is feasible and potentially effective, provided that BCMA expression is retained [32]. With the anticipated expansion of cilta-cel into earlier lines, and the ongoing evaluation of frontline application in the CARTITUDE-5 and CARTITUDE-6 trials, a second CAR T-cell therapy at relapse may become a viable treatment sequencing strategy.

An additional practical consideration for earlier-line application is the potential for improved manufacturing feasibility and more predictable treatment timelines [33]. In the Late group, five patients died between apheresis and infusion, highlighting the urgent unmet need to streamline the manufacturing process, particularly for patients with rapidly progressive disease. Although turnaround times were similar across groups, patients in earlier lines are inherently less likely to experience fatal disease acceleration during the manufacturing interval, a consideration that increasingly supports early CAR T-cell therapy deployment.

This study has several limitations inherent to its registry-based design. First, median follow-up was shorter in the Early-line than in the Late-line cohort, an imbalance that reflects the staggered regulatory approval of cilta-cel rather than differential censoring; nonetheless, follow-up in the early-line cohort is sufficient to capture the early-relapse hazard that defines short-term outcomes after BCMA-directed CAR T-cell therapy. Second, the full IMS-IMWG 2024 and R2-ISS high-risk algorithms could not be reconstructed because clonal cell fraction of del(17p), *TP53* mutation status, del(1p32), the distinction between gain and amplification of 1q21, LDH, and β2-microglobulin are not captured by the registry minimum dataset, and the cytogenetic or FHR stratification presented here should therefore be interpreted as an approximation of these contemporary frameworks. Third, missingness for key baseline variables was non-negligible and, although broadly balanced between the Early- and Late-line cohorts, restricted stratified analyses to complete cases and leaves residual potential for informative missingness that cannot be excluded. Furthermore, the registry captures adverse events at reporting-window resolution but does not record day-level onset, event duration, or structured management data, precluding a formal time-to-event analysis of these toxicities, suggesting cautious interpretation of these results. Fourth, data on CAR T-cell expansion kinetics, which may be more favorable in earlier lines and are relevant both for efficacy and for the risk of hyperexpansion-associated NiNTs, were not available. While earlier application may theoretically be associated with fitter T cells and greater expansion potential, we did not observe an increased NiNT signal in the Early group. However, given the later regulatory approval in the earlier-line setting, follow-up remains shorter and longer observation is warranted. Additionally, molecular minimal residual disease assessments, and soluble BCMA levels were not systematically collected. Finally, the rapidly evolving treatment landscape must be acknowledged. The recent results of the MajesTEC-3 trial, which demonstrated an 83% reduction in the risk of progression or death with teclistamab plus daratumumab in a similar second-line population [34], introduce bispecific antibody-based combinations as a potentially competing strategy in the same therapeutic space. Defining the optimal sequencing of CAR T-cell therapy and bispecific antibodies in the earlier-line setting will require prospective, randomized comparison.

Beyond the scope of the present analysis, structured prospective capture of rare and delayed toxicities, including IEC-enterocolitis, secondary CAR-positive T-cell lymphomas, and clonal myeloid events represents a critical unmet need that cannot be addressed within the confines of clinical trials. Given the substantial phenotypic and biological overlap between these entities, most notably between IEC-enterocolitis and clonal CAR-T cell proliferations in refractory cases, harmonized real-world registries with integrated clinical, molecular, and histopathological adjudication will be essential to delineate their true incidence, underlying mechanisms, and optimal management. Expanding the German CAR-T registry toward systematic longitudinal capture of these late events complemented by the prospective collection of peripheral blood, bone marrow, and tissue biospecimen to enable granular molecular, single-cell, and clonal analyses therefore constitutes a key future direction of our ongoing work.

In summary, this nationwide registry analysis demonstrates that cilta-cel administered in earlier lines of therapy in routine clinical practice is safe and effective, with outcomes consistent with the CARTITUDE-4 trial. These data support the evolving paradigm of deploying cilta-cel as early as first relapse and provide a framework for identifying patients who may derive the greatest benefit from this therapy.

## Supplementary Information

Below is the link to the electronic supplementary material.

Supplementary Material 1.

## Data Availability

The datasets used and/or analysed during the current study are available from the corresponding author on reasonable request.

## References

[1] San-Miguel J, Dhakal B, Yong K, Spencer A, Anguille S, Mateos M-V, et al. Cilta-cel or Standard Care in Lenalidomide-Refractory Multiple Myeloma. N Engl J Med. 2023;389:335–47. 37272512 10.1056/NEJMoa2303379

[2] Einsele H, San-Miguel J, Dhakal B, Touzeau C, Leleu X, Donk NW, van de, et al. Cilta-cel in lenalidomide-refractory multiple myeloma (CARTITUDE-4): an updated analysis including overall survival from an open-label, multicentre, randomised, phase 3 trial. Lancet Oncol. 2026;27:254–68. 41519141 10.1016/S1470-2045(25)00653-9

[3] Dhakal B, Iida S, Sidiqi MH, Zamagni E, Yoon S-S, Callander N, et al. Effectiveness of bridging therapy corresponds to improved outcomes after ciltacabtagene autoleucel: Phase 3 CARTITUDE-4 study of patients with relapsed, lenalidomide-refractory multiple myeloma. Blood. 2025;146:2215.

[4] Hansen DK, Peres LC, Dima D, Richards A, Shune L, Afrough A, et al. Comparison of Standard-of-Care Idecabtagene Vicleucel and Ciltacabtagene Autoleucel in Relapsed/Refractory Multiple Myeloma. J Clin Oncol Off J Am Soc Clin Oncol. 2025;43:1597–609. 10.1200/JCO-24-01730 PMC12037312 39965175

[5] Merz M, Albici A-M, von Tresckow B, Rathje K, Fenk R, Holderried T, et al. Idecabtagene vicleucel or ciltacabtagene autoleucel for relapsed or refractory multiple myeloma: An international multicenter study. HemaSphere. 2025;9:e70070. 39822585 10.1002/hem3.70070 PMC11735948

[6] Merz M, Gagelmann N, Smaili S, Flossdorf S, Sauer S, Scheid C, et al. Remission conversion drives outcomes after CAR T-cell therapy for multiple myeloma: a registry analysis from the DRST. Blood. 2025;146:1677–86. 40504993 10.1182/blood.2025028330

[7] Kumar S, Paiva B, Anderson KC, Durie B, Landgren O, Moreau P, et al. International Myeloma Working Group consensus criteria for response and minimal residual disease assessment in multiple myeloma. Lancet Oncol. 2016;17:e328–46. 27511158 10.1016/S1470-2045(16)30206-6

[8] Lee DW, Santomasso BD, Locke FL, Ghobadi A, Turtle CJ, Brudno JN, et al. ASTCT Consensus Grading for Cytokine Release Syndrome and Neurologic Toxicity Associated with Immune Effector Cells. Biol Blood Marrow Transpl J Am Soc Blood Marrow Transpl. 2019;25:625–38. 10.1016/j.bbmt.2018.12.758 PMC12180426 30592986

[9] Martin T, Usmani SZ, Berdeja JG, Agha M, Cohen AD, Hari P, et al. Ciltacabtagene Autoleucel, an Anti-B-cell Maturation Antigen Chimeric Antigen Receptor T-Cell Therapy, for Relapsed/Refractory Multiple Myeloma: CARTITUDE-1 2-Year Follow-Up. J Clin Oncol Off J Am Soc Clin Oncol. 2023;41:1265–74. 10.1200/JCO.22.00842 PMC9937098 35658469

[10] Berdeja JG, Madduri D, Usmani SZ, Jakubowiak A, Agha M, Cohen AD, et al. Ciltacabtagene autoleucel, a B-cell maturation antigen-directed chimeric antigen receptor T-cell therapy in patients with relapsed or refractory multiple myeloma (CARTITUDE-1): a phase 1b/2 open-label study. Lancet Lond Engl. 2021;398:314–24. 10.1016/S0140-6736(21)00933-8 34175021

[11] Jagannath S, Martin TG, Lin Y, Cohen AD, Raje N, Htut M, et al. Long-Term (≥ 5-Year) Remission and Survival After Treatment With Ciltacabtagene Autoleucel in CARTITUDE-1 Patients With Relapsed/Refractory Multiple Myeloma. J Clin Oncol Off J Am Soc Clin Oncol. 2025;43:2766–71. 10.1200/JCO-25-00760 PMC12393059 40459151

[12] Sebastian T, Hashmi H, Firestone R, Jurgens E, Rajeeve S, Farzana T, et al. Functionally high-risk disease is associated with inferior outcomes after chimeric antigen receptor T-cell therapy for relapsed refractory multiple myeloma. Blood. 2025;146:2291.

[13] Parekh S, Li K, Van De Donk NWCJ, Lin Y, Martin T, Han S, et al. Earlier use of ciltacabtagene autoleucel (cilta-cel) is associated with better immune fitness and stronger immune effects as shown by correlative analysis of peripheral blood and the bone marrow tumor microenvironment (TME) from the CARTITUDE-4 study. Blood. 2025;146:92.

[14] Richter J. Like a bridge over troubled water: keeping the myeloma down en route to CAR-T. Blood Cancer J. 2024;14:64. 38609377 10.1038/s41408-024-01049-z PMC11015014

[15] Cohen AD, Parekh S, Santomasso BD, Gállego Pérez-Larraya J, van de Donk NWCJ, Arnulf B, et al. Incidence and management of CAR-T neurotoxicity in patients with multiple myeloma treated with ciltacabtagene autoleucel in CARTITUDE studies. Blood Cancer J. 2022;12:32. 10.1038/s41408-022-00629-1 PMC8873238 35210399

[16] Ho M, Paruzzo L, Noll JH, Stella F, Devi P, Ndeupen S, et al. CD4 + T cells mediate CAR-T cell-associated immune-related adverse events after BCMA CAR-T cell therapy. Nat Med. 2026;32:702–16. 41540109 10.1038/s41591-025-04121-8 PMC13070021

[17] Rade M, Fandrei D, Kreuz M, Seiffert S, Grahnert A, Friedrich M et al. A longitudinal single-cell atlas to predict outcome and toxicity after BCMA-directed CAR T cell therapy in multiple myeloma. Cancer Cell 2026;44(3):586–603. 10.1016/j.ccell.2025.10.014 41349540

[18] Hosoya H, Velayati A, Dima D, Jensen A, Portuguese A, Hovanky V, et al. Rapid peak CAR-T expansion is associated with delayed neurotoxicity following ciltacabtagene autoleucel in multiple myeloma. Blood. 2025;146:96. 10.1182/blood.2025032844 42418682

[19] Lim KJC, Parrondo RD, Chhabra S, Dooley K, De Menezes Silva Corraes A, Gertz MA, et al. Investigating the association between peak post-infusion absolute lymphocyte count (ALC) and delayed toxicity in myeloma (MM) patients (pts) receiving cilta-cel. J Clin Oncol. 2025;43:7522–7522.

[20] Jurgens EM, Mitra S, Herrera K, Nemirovsky D, Bready B, Derkach A et al. Robust CD4 + CAR T cell Expansion Is Associated with Non-ICANS Neurotoxicities Following Ciltacabtagene Autoleucel. MedRxiv Prepr Serv Health Sci 2025;2025.10.28.25338924.

[21] Rodríguez-Otero P, Sidana S, Kouwenhoven M, Schecter JM, Lendvai N, De Braganca KC, et al. Clinical experience with cranial nerve palsy in patients infused with ciltacabtagene autoleucel for the treatment of relapsed/refractory MM in CARTITUDE-1, -2, and – 4. Blood Cancer J. 2025;15:209. 41298382 10.1038/s41408-025-01410-w PMC12658040

[22] Zanwar S, Sidana S, Shune L, Puglianini OC, Pasvolsky O, Gonzalez R, et al. Impact of extramedullary multiple myeloma on outcomes with idecabtagene vicleucel. J Hematol OncolJ Hematol Oncol. 2024;17:42. 38845015 10.1186/s13045-024-01555-4 PMC11157748

[23] Gagelmann N, Dima D, Merz M, Hashmi H, Ahmed N, Tovar N, et al. Development and Validation of a Prediction Model of Outcome After B-Cell Maturation Antigen-Directed Chimeric Antigen Receptor T-Cell Therapy in Relapsed/Refractory Multiple Myeloma. J Clin Oncol Off J Am Soc Clin Oncol. 2024;42:1665–75. 10.1200/JCO.23.02232 PMC11095856 38358946

[24] Born P, Fandrei D, Wang SY, Perez-Fernandez C, Fischer L, Ussmann J, et al. Prognostic significance of PET/CT for CAR T cell therapy in relapsed/refractory multiple myeloma. HemaSphere. 2025;9:e70159. 40519771 10.1002/hem3.70159 PMC12167625

[25] van de Donk NWCJ, Moreau P, San-Miguel JF, Mateos M-V, Dimopoulos MA, Zweegman S, et al. Optimising T-cell immunotherapy in patients with multiple myeloma: practical considerations from the European Myeloma Network. Lancet Haematol. 2025;12:e635–49. 40580975 10.1016/S2352-3026(25)00117-6

[26] van de Donk NWCJ, Sonneveld P, Einsele H. Dual-antigen-targeting T-cell immunotherapies in MM: circumventing tumor heterogeneity and preventing antigen escape. Blood 2026;blood.2025032536. 10.1182/blood.2025032536 41610427

[27] Cohen YC, Magen H, Gatt M, Sebag M, Kim K, Min C-K, et al. Talquetamab plus Teclistamab in Relapsed or Refractory Multiple Myeloma. N Engl J Med. 2025;392:138–49. 39778168 10.1056/NEJMoa2406536

[28] Cohen A, Susanibar-Adaniya S, Garfall A, Vogl D, Kapur S, Waxman A, et al. Phase 2 study of cevostamab consolidation following BCMA CAR T cell therapy: preliminary safety, efficacy, and correlative data from the STEM (Sequential T Cell-Engagement for Myeloma) trial. Blood. 2025;146:699.

[29] Dhakal B, Akhtar OS, Fandrei D, Jensen A, Banerjee R, Pan D, et al. Sequential targeting in multiple myeloma: talquetamab, a GPRC5D bispecific antibody, as a bridge to BCMA CAR-T therapy. Blood. 2025;146:2063–72. 40749169 10.1182/blood.2025029773

[30] Fandrei D, Seiffert S, Rade M, Rieprecht S, Gagelmann N, Born P, et al. Bispecific Antibodies as Bridging to BCMA CAR-T Cell Therapy for Relapsed/Refractory Multiple Myeloma. Blood Cancer Discov. 2025;6:38–54. 39441177 10.1158/2643-3230.BCD-24-0118 PMC11707513

[31] Schmidt TM, Fonseca R, Usmani SZ. Chromosome 1q21 abnormalities in multiple myeloma. Blood Cancer J. 2021;11:83. 33927196 10.1038/s41408-021-00474-8 PMC8085148

[32] Costa LJ, Banerjee R, Mian H, Weisel K, Bal S, Derman BA, et al. International myeloma working group immunotherapy committee recommendation on sequencing immunotherapy for treatment of multiple myeloma. Leukemia. 2025;39:543–54. 39870767 10.1038/s41375-024-02482-6 PMC11879857

[33] Bowden R, Araque J, Choudhary G, Kallenbach L, Alegria V, Costa O, et al. Ciltacabtagene autoleucel out-of-specification manufacturing outcomes improve with earlier lines of therapy. Blood. 2025;146:2411.

[34] Costa LJ, Bahlis NJ, Perrot A, Nooka AK, Lu J, Pawlyn C, et al. Teclistamab plus daratumumab in relapsed or refractory multiple myeloma. N Engl J Med. 2026;394:739–52. 41363801 10.1056/NEJMoa2514663 PMC13218738

