# Supplementary material for: Standard-of-care ciltacabtagene autoleucel in earlier versus later lines of therapy for relapsed or refractory multiple myeloma: a nationwide registry analysis
Source: J Hematol Oncol. 2026 May 20;19:46. doi: 10.1186/s13045-026-01806-6 (PMC13307700; doi:10.1186/s13045-026-01806-6)
Supplement: Supplementary file 1 — Supplementary Material 1. [file 13045_2026_1806_MOESM1_ESM.docx]

Supplement for:

**Standard-of-care ciltacabtagene autoleucel in earlier versus later lines of therapy for relapsed or refractory multiple myeloma: a nationwide registry analysis**

Gagelmann et al.

**Content**

**Supplementary Methods and Results – pp. 3-5**

**Supplementary Tables – p. 6**

**Supplementary Figures – pp. 7-8**

**DRST members contributing to this study – p. 9**

**Supplementary Methods and Results**

**Probabilistic identification of functional high-risk disease.**

The IMWG-consensus definition of functional high-risk (FHR) multiple myeloma — progression-free survival after frontline therapy (PFS1) <18 months — cannot be computed directly from DRST because the date of frontline therapy initiation and the date of first progression are not captured as separable fields. We therefore used a two-step probabilistic strategy to identify FHR patients across the entire cohort.

*Training label.* Among the patients with documented prior autologous stem-cell transplantation (ASCT) – the canonical anchor for end-of-frontline therapy in transplant-eligible RRMM – we derived a registry-observable reference label by dichotomizing the ASCT-to-cilta-cel interval at 24 months. This threshold places a strict upper bound on PFS1: any patient with ASCT-to-cilta-cel <24 months must have had PFS1 below 24 months. For the Late-group training label, we used an extended definition (ASCT-to-cilta-cel <36 months OR mean months-per-post-ASCT-line <9 months) to capture FHR biology in patients whose originally rapid PFS1 was followed by multiple subsequent lines before CAR T-cell therapy.

*Group-specific models.* Preliminary analysis using a cohort-wide model revealed that the dominant predictor in the general logistic regression was confounded in the Late-line group, where heavily pretreated patients with originally rapid PFS1 but multiple subsequent lines have long total observation times that mask their underlying FHR biology. We therefore fitted two L2-penalized logistic regression models with class-balancing. For the Early group, a general model used 12 baseline predictors (diagnosis-to-infusion interval, number of prior lines, ISS stage, extramedullary disease, composite high-risk cytogenetics, ECOG ≥2, refractory disease status at infusion, and age at infusion). For the Late group, a velocity-based model used 16 predictors prioritizing features that capture early disease dynamics independent of total observation time: lines-per-year since diagnosis, mean months-per-line, number of prior lines, ISS stage, extramedullary disease, composite high-risk cytogenetics, ECOG ≥2, refractory disease status at infusion, prior CAR T-cell exposure (ide-cel), prior CD38 exposure, and age at infusion. Continuous predictors were z-score standardized; missing covariates were median-imputed.

*Model performance.* The general model achieved a 5-fold stratified cross-validated area under the ROC curve (AUC) of 0.89; the Late-specific velocity model achieved a cross-validated AUC of 0.93. Internal calibration within the training sets was adequate, with median posterior probability of 0.83 in observed FHR patients versus 0.16 in non-FHR patients for the general model.

*Application and threshold calibration.* The fitted general model was applied to all Early-group patients and its classification threshold calibrated to a 20% target prevalence in the overall cohort, centered on the published real-world range for RRMM/CAR-T cohorts (15-30%; Corre et al., *Leukemia* 2020; Hashmi et al., *Blood Cancer Journal* 2026). The Late-specific model was applied to all Late-group patients and its threshold calibrated to a strict 15% target prevalence within the Late group, deliberately chosen to isolate the biologically most distinct FHR subgroup – the concentration corresponding to the PFS1 <18-month threshold reported in newly-diagnosed RRMM (Corre 2020) – rather than the ~25-30% enrichment typical of CAR T-cell referral cohorts (Hashmi et al.). This strict calibration reflects the recognition that CAR T-cell cohorts are inherently enriched for FHR biology through clinician-driven referral patterns, and that a conservative threshold yields the cleanest biological separation for identifying residual prognostic signal.

*Sensitivity analyses.* Sensitivity analyses varying the Late-group prevalence target between 15% and 35% showed a monotonically attenuating effect size (HR 1.69 at 15%, 1.24 at 20%, 1.26 at 25%, 1.11 at 30%, 1.10 at 35%) supporting the 15% calibration as the threshold at which the FHR signal is most clearly separated from general treatment-refractoriness phenotypes. Directional consistency was preserved across all tested thresholds. Analogous sensitivity analyses for the Early-group model showed directional consistency of the null finding (no significant residual penalty) across thresholds of 15-30%.

**Supplementary Tables**

**Table S1. Bridging therapy regimens administered prior to ciltacabtagene autoleucel infusion, stratified by treatment line.**

*Counts and percentages are reported for the 159 of 601 patients (26%) in whom regimen-level bridging therapy details were prospectively captured in DRST.*

| **Bridging therapy class** | **Overall**  **(N=159)** | **Early**  **(N=47)** | **Late**  **(N=112)** |
| --- | --- | --- | --- |
| **Proteasome inhibitor-based regimens** |  |  |  |
| Any PI-containing regimen | 78 (49%) | 25 (53%) | 53 (47%) |
| Carfilzomib-based | 58 (36%) | 20 (43%) | 38 (34%) |
| Bortezomib-based | 19 (12%) | 5 (11%) | 14 (12%) |
| **Anti-CD38 monoclonal antibody-containing** |  |  |  |
| Daratumumab or isatuximab | 56 (35%) | 24 (51%) | 32 (29%) |
| **Immunomodulatory drug (IMiD)-containing** |  |  |  |
| Lenalidomide, pomalidomide, or thalidomide | 49 (31%) | 20 (43%) | 29 (26%) |
| **SLAMF7-directed monoclonal antibody** |  |  |  |
| Elotuzumab-containing | 24 (15%) | 11 (23%) | 13 (12%) |
| **Classical cytotoxic chemotherapy** |  |  |  |
| Alkylator or combination chemotherapy | 26 (16%) | 6 (13%) | 20 (18%) |
| **Radiotherapy as part of bridging** |  |  |  |
| Any radiotherapy | 61 (10%) | 10 (6%) | 51 (12%) |
| **T-cell–redirecting and other novel agents** |  |  |  |
| Talquetamab | 35 (22%) | 0 | 35 (31%) |
| Teclistamab | 1 (<1%) | 0 | 1 (<1%) |
| Selinexor-containing regimens | 6 (4%) | 0 | 6 (5%) |
| Venetoclax-containing regimens | 4 (3%) | 0 | 4 (4%) |

*Abbreviations:* ADC, antibody–drug conjugate; BCMA, B-cell maturation antigen; BsAb, bispecific antibody; EMA, European Medicines Agency; FDA, US Food and Drug Administration; GPRC5D, G-protein–coupled receptor family C group 5 member D; IMiD, immunomodulatory drug; MM, multiple myeloma; PI, proteasome inhibitor; SLAMF7, signaling lymphocytic activation molecule family member 7.

**Supplementary Figures.**

**Figure S1. PFS by functional high risk (FHR) status.**

Kaplan-Meier estimates of PFS in (A) the Early-line cohort (1-3 prior lines) and (B) the Late-line cohort (>3 prior lines), stratified by predicted FHR status. Shaded bands represent 95% confidence intervals (log-log transformation); vertical tick marks denote censored observations. 6- and 12-month landmark PFS estimates with 95% confidence intervals are shown in each panel, with numbers at risk below.

**
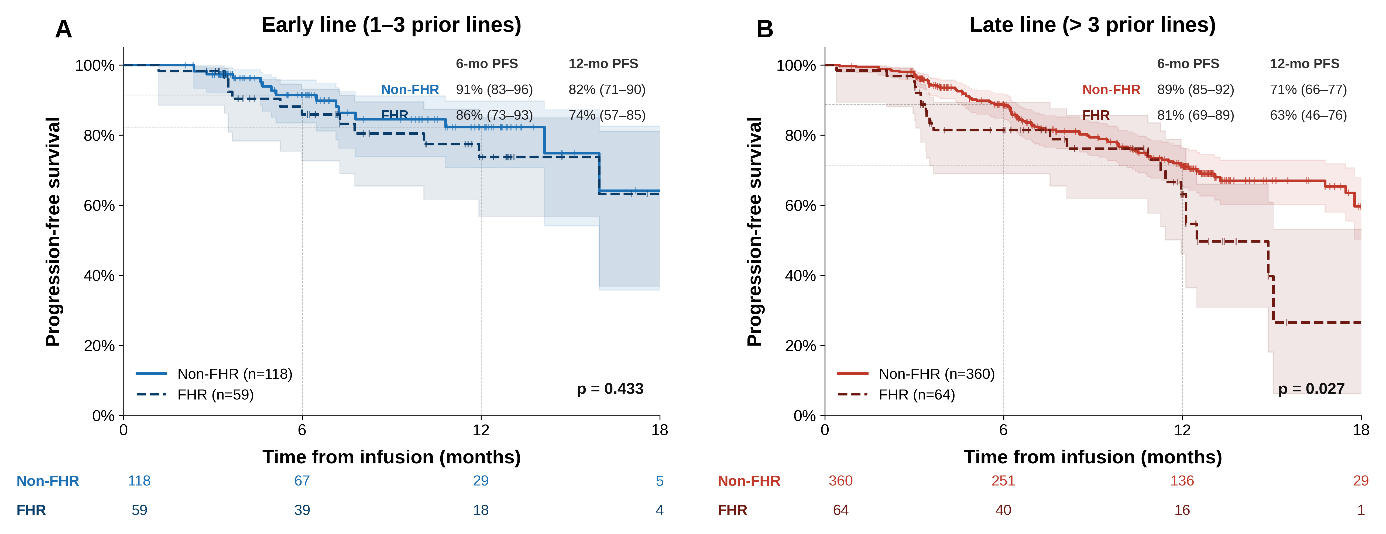
**

**Figure S2. Bridging outcomes.**

Kaplan-Meier estimates of PFS in (A) the Early-line bridging cohort (1-3 prior lines; n=47), (B) the Late-line bridging cohort (>3 prior lines; n=112), and (C) the subgroup receiving talquetamab-containing bridging regardless of original line assignment (n=35). Shaded bands represent 95% confidence intervals (log-log transformation); vertical tick marks denote censored observations. 6- and 12-month landmark PFS estimates with 95% confidence intervals are shown in each panel, with numbers at risk below.

**
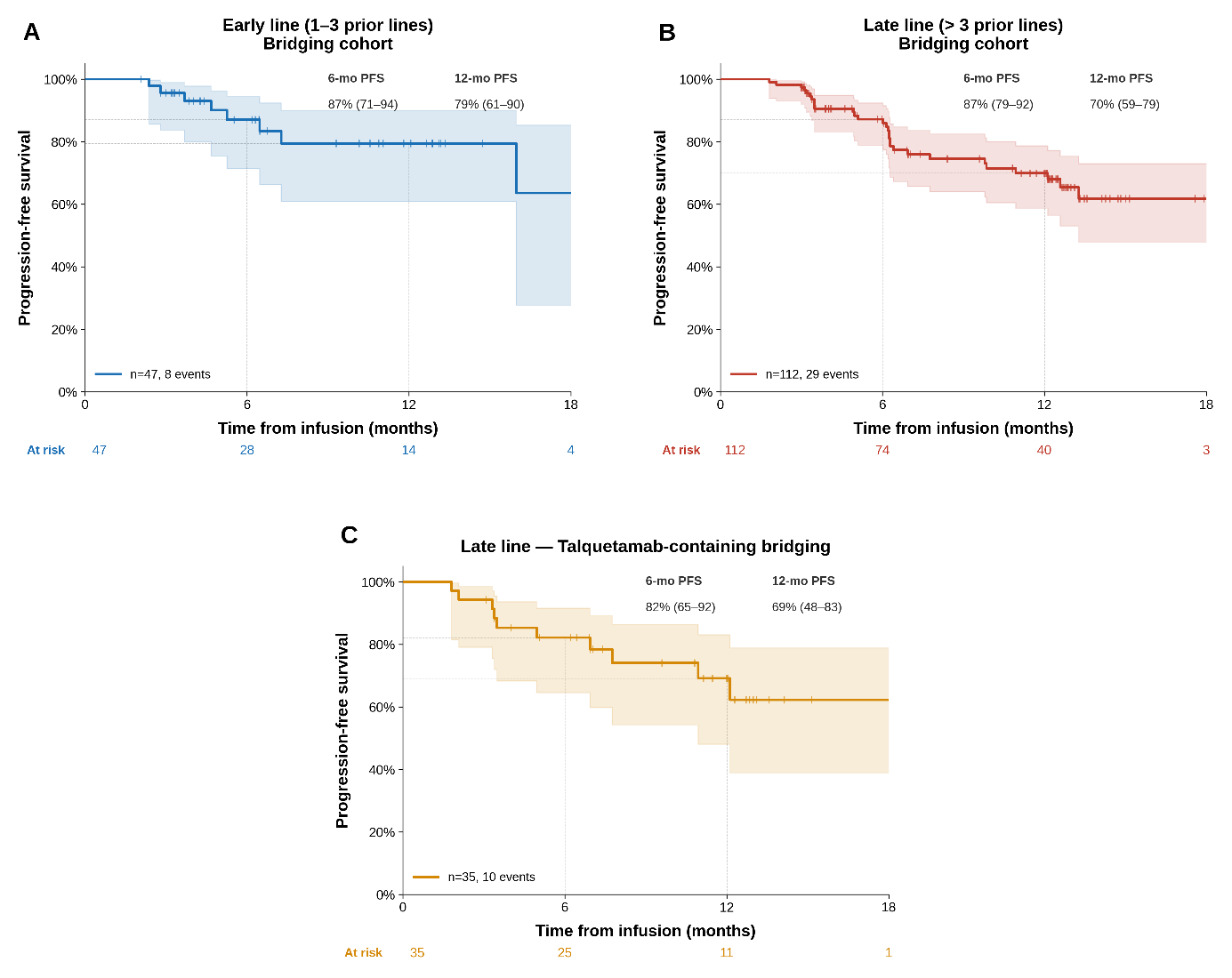
**

**DRST members contributing to this study**

Universitätsklinikum Würzburg, 85 patients

Universitätsklinikum Leipzig, 76 patients

Universitätsklinikum Köln, 70 patients

Charité - Universitätsmedizin Berlin, 68 patients

Universitätsklinikum Heidelberg, 57 patients

Universitätsklinikum Freiburg, 42 patients

Universitätsklinikum Essen, 32 patients

Universitätsklinikum Carl Gustav Carus a. d. TU Dresden, 31 patients

Universitätsklinikum Hamburg-Eppendorf, 30 patients

Universitätsklinikum Tübingen, 25 patients

Universitätsklinikum Regensburg, 18 patients

Universitätsklinikum Schleswig-Holstein / Campus Kiel, 14 patients

Universitätsklinikum Hamburg-Eppendorf, Klinik für Stammzelltransplantation, 12 patients

TUM Universitätsklinikum, Klinikum rechts der Isar, 11 patients

Med. Hochschule Hannover, 9 patients

Knappschaft Kliniken Universitätsklinikum Bochum, 7 patients

Klinikum Chemnitz gGmbH, 6 patients

Robert-Bosch-Krankenhaus, 4 patients

Asklepios Klinik St. Georg, 2 patients

Univ.-Klinikum Gießen und Marburg GmbH, 2 patients

Universitätsklinikum Halle (Saale), 1 patient

Universitätsklinikum Magdeburg A. ö. R., 1 patient

Klinikum Nürnberg Nord, 1 patient

Universitätsklinikum Erlangen, 1 patient
